# Involvement of NpdA, a Putative 2-Nitropropane Dioxygenase, in the T3SS Expression and Full Virulence in *Ralstonia solanacearum* OE1-1

**DOI:** 10.3389/fmicb.2017.01990

**Published:** 2017-10-11

**Authors:** Weiqi Zhang, Jing Li, Yu Tang, Kai Chen, Xiaojun Shi, Kouhei Ohnishi, Yong Zhang

**Affiliations:** ^1^College of Resources and Environment, Southwest University, Chongqing, China; ^2^The Ninth People’s Hospital of Chongqing, Chongqing, China; ^3^Department of Pharmacognosy, College of Pharmacy, Third Military Medical University, Chongqing, China; ^4^Research Institute of Molecular Genetics, Kochi University, Kochi, Japan

**Keywords:** *Ralstonia solanacearum*, type III secretion system, Hrp regulon, Pathogenesis, NPD

## Abstract

Previously, we isolated several genes that potentially affected the expression of type III secretion system (T3SS) in *Ralstonia solanacearum* OE1-1. Here, we focused on the *rsp0316*, which encodes a putative 2-nitropropane dioxygenase (hereafter designated NpdA). The deletion of *npdA* substantially reduced the T3SS expression and virulence in OE1-1, and the complementation with functional NpdA could completely restore its reduced T3SS expression and virulence to that of wild type. The NpdA was highly conserved among diverse *R. solanacearum* species and the NpdA-dependent expression of T3SS was not specific to OE1-1 strain, but not the virulence. The NpdA was important for the T3SS expression *in planta*, while it was not required for the bacterial growth *in planta*. Moreover, the NpdA was not required for the elicitation of hypersensitive response (HR) of *R. solanacearum* strains in tobacco leaves. The T3SS in *R. solanacearum* is directly controlled by the AraC-type transcriptional regulator HrpB and regulated by a complex regulation network. The NpdA affected the T3SS expression mediated with HrpB but through some novel pathway. All these results from genetic studies demonstrate that NpdA is a novel factor for the T3SS expression in diverse *R. solanacearum* species in medium, but specifically for the T3SS expression in strain OE1-1 *in planta*. And the NpdA-dependent expression of T3SS *in planta* plays an important role in pathogenicity of *R. solanacearum* OE1-1 in host plants.

## Introduction

The syringe-like type III secretion system (T3SS) is conserved in many bacterial pathogens of animals, plants, and insects and plays central roles in their pathogenicity (Hueck, 1998; Galán and Wolf-Watz, 2006). The bacteria use the T3SS to interact with host cells and inject virulence factors, so-called type III effectors (T3Es), into the host cytosol to subvert host functions (Angot et al., 2006; Jones and Dangl, 2006). In some bacterial plant pathogens, including the *Ralstonia solanacearum*, the T3SS is encoded by hypersensitive response and pathogenicity (*hrp*) genes, which are approximately 20 genes clustered together into a regulon (Arlat et al., 1992; Van Gijsegem et al., 1995). *R. solanacearum* is a soil-born Gram-negative vascular bacterium and can devastate more than 200 plant species worldwide (Genin, 2010). This results in the serious loss in many economically important plants every year, and until recently, there has been no general effective control for this bacterial wilt disease.

The T3SS in *R. solanacearum* is not constitutively expressed under any conditions. Its expression is not activated until the bacteria have contact with host signals (Aldon et al., 2000). The T3SS is not expressed under nutrient-rich conditions, but it is activated under nutrient-poor conditions, which might mimic the conditions of intercellular spaces in host cells. Moreover, when the bacteria directly contact host plants, the expression of the T3SS was induced to a 10- to 20-fold higher level than that under nutrient-poor conditions (Marenda et al., 1998; Mukaihara et al., 2004; Yoshimochi et al., 2009b; Zhang et al., 2011). *R. solanacearum* has a large repertoire of T3Es (averaged more than 70 T3Es per strain), and most of them play important roles in the interaction between *R. solanacearum* and host cells (Coll and Valls, 2013; Peeters et al., 2013). The T3SS and entire T3Es are directly controlled by the master regulator HrpB, which is an AraC family transcriptional regulator and is essential for the pathogenicity of *R. solanacearum* (Arlat et al., 1992; Genin et al., 1992; Vasse et al., 2000; Mukaihara et al., 2004, 2010). The expression of *hrpB* is triggered by plant signals or other related signals. These signals are recognized and transferred to HrpB through the complex signaling cascade PrhA–PrhR/I–PrhJ-HrpG or some unknown pathway (Brito et al., 2002; Cunnac et al., 2004; Valls et al., 2006). Moreover, the expression of *hrpB* is positively regulated in a parallel way by both HrpG and prhG, which are close paralogs and response regulators of the OmpR/PhoB family of two-component system (Plener et al., 2010; Zhang et al., 2013). However, the quorum sensing-dependent regulator PhcA regulates the expression of *hrpG* and *prhG* in an opposite ways. PhcA negatively regulates *hrpG* expression indirectly (Yoshimochi et al., 2009a) but positively regulates *prhG* expression (Zhang et al., 2013). *R. solanacearum* might dynamically activate *hrpB* expression through HrpG and PrhG in a cell density-dependent manner (Zhang et al., 2013). Generally, *R. solanacearum* uses a complex regulation network to globally regulate the expression of the T3SS and promote disease development in host plants, but this network needs to be further elucidated (Valls et al., 2006; Hikichi et al., 2007).

In our study, the T3SS expression in OE1-1 was monitored with a *popA-lacZYA* fusion, which combining the promoter of *popA* to promoterless *lacZYA* (a schematic is available as Supplementary Figure S1 in Zhang et al., 2013) and it exhibited almost identical expression profiles to that of T3SS under different conditions. Moreover, the generation of *popA-lacZYA* did not affect the bacterial growth and virulence of OE1-1 in host plants. Here, we focused on a novel candidate, Rsp0316 (prh24 in Zhang et al., 2013), which was isolated from the Japanese *R. solanacearum* strain OE1-1 with transposon mutagenesis (Zhang et al., 2013). The *popA* expression in this Tn5 mutant was substantially reduced, suggesting that the Rsp0316 might be a positive effector for the T3SS expression in *R. solanacearum* and hence related to its virulence. Rsp0316 encodes a putative 2-nitropropane dioxygenase (NPD) and is highly conserved in all *Ralstonia* species and other bacteria (hereafter designated NpdA). NPDs are known to participate in nitrogen metabolism, but the involvement of NPDs in the virulence of pathogens has rarely been investigated (Gorlatova et al., 1998; Francis et al., 2005). Here, we characterized the impact of NpdA on the T3SS expression and the virulence of *R. solanacearum* in host plants and demonstrate that the NpdA is required for the T3SS expression in diverse *R. solanacearum* species in medium but specifically for the T3SS expression *in planta* and full virulence of *R. solanacearum* OE1-1 in host plants.

## Materials and Methods

### Bacterial Strains and Culture Conditions

*Ralstonia solanacearum* strains used in this study were listed in **Table 1**. OE1-1 strain (phylotype I, race 1, biovar 3) is virulent in tomato and tobacco plants (Kanda et al., 2003). RS1002 (Mukaihara et al., 2004) and GMI1000 (Salanoubat et al., 2002) strain (phylotype I, race 1, biovar 4) are virulent in tomato plants but elicit hypersensitive response (HR) in tobacco leaves. *R. solanacearum* was grown at 28°C in rich B medium or in *hrp-*inducing medium (hydroponic plant culture medium containing 2% sucrose) (Yoshimochi et al., 2009b). *Escherichia coli* DH12S and S17-1 (Simon et al., 1983) were grown in Luria-Bertani (LB) medium at 37°C and used for plasmid construction and conjugational transfer, respectively. Antibiotics were added into the media at the following concentrations: ampicillin (Ap), 100 μg ml^-1^; gentamicin (Gm), 20 μg ml^-1^; kanamycin (Km), 50 μg ml^-1^; polymyxin B (PB), 50 μg ml^-1^.

**Table 1:** *Ralstonia solanacearum* stains used in this study.

Strain	Relative characteristics	Reference
OE1-1	Wild-type, race 1, biovar 3	Kanda et al., 2003
RK5043	OE1-1 *phcA-lacZYA*	Yoshimochi et al., 2009b
RK5046	OE1-1 *hrpB-lacZYA*	Yoshimochi et al., 2009b
RK5050	OE1-1 *popA-lacZYA*	Yoshimochi et al., 2009b
RK5120	OE1-1 *hrpG-lacZYA*	Yoshimochi et al., 2009b
RK5124	OE1-1 *prhJ-lacZYA*	Yoshimochi et al., 2009b
RK5130	OE1-1 *prhIR-lacZYA*	Yoshimochi et al., 2009b
RK5134	OE1-1 *prhA-lacZYA*	Yoshimochi et al., 2009b
RK5212	OE1-1 *prhG-lacZYA*	Zhang et al., 2013
RK5619	OE1-1 *prhN-lacZYA*	Zhang et al., 2015
RK5639	OE1-1 *phcA-lacZYA*, Δ*npdA*	This work
RK5630	OE1-1 *hrpB-lacZYA*, Δ*npdA*	This work
RK5628	OE1-1 *popA-lacZYA*, Δ*npdA*	This work
RK5637	OE1-1 *hrpG-lacZYA*, Δ*npdA*	This work
RK5634	OE1-1 *prhJ-lacZYA*, Δ*npdA*	This work
RK5641	OE1-1 *prhIR-lacZYA*, Δ*npdA*	This work
RK5643	OE1-1 *prhA-lacZYA*, Δ*npdA*	This work
RK5644	OE1-1 *prhG-lacZYA*, Δ*npdA*	This work
RK5632	OE1-1 *prhN-lacZYA*, Δ*npdA*	This work
RK7007	RK5628 (pUCnpdA)	This work
RS1002	Wild-type, race 1, biovar 4	Mukaihara et al., 2004
RK10001	RS1002, *popA-lacZYA*	Zhang et al., 2013
RK10013	RK10001, Δ*npdA*	This work
RK10015	RK10010 (Δ*npdA*, pUCnpdA)	This work
GMI1000	Wild-type, race 1, biovar 4	Salanoubat et al., 2002
GF0021	GMI1000, *popA-lacZYA*,Δ*npdA*	This work


### Construction of *npdA* in-Frame Deletion Mutants

In this study, the *npdA* in-frame deletion mutants were generated by pK18mobsacB- based homolog recombination as previously described (Zhang et al., 2015). Primer pairs 0316A1B (CATGGATCCATCAACCGTTCGAAGGAGA) with 0316B1C (TCAACGGAGACTTGTGCCCCACCCGTTGCATCCGCG) and 0316A2C (CGCGGATGCAACGGGTGGGGCACAAGTCTCCGTTGA) with 0316B2H (CATAAGCTTCCATCTGGACCTTCAGGT) were used for plasmid pK18dnpdA construction with the OE1-1 genomic DNA as PCR template. After validating the sequence, pK18dnpdA was transferred from *E. coli* S17-1 into the corresponding *R. solanacearum* strains, and *npdA* mutants were generated (listed in **Table 1**).

### Complementation Analysis

The complementation was performed by using the pUC18-mini-Tn7T-Gm-mediated site-specific chromosome integration system (Choi et al., 2005) with some modification (Zhang et al., 2015). Primers pairs 0316A1B and 0316B3H (CATAAGCTTCAGGCCGTCGTCGCCGAAG) were used for the pUCnpdA construction with the OE1-1 genomic DNA as the template, which contained an approximately 500 bp-upstream region of *npdA* and empirically harbored its native promoter. The complemented *npdA* was specifically integrated into *R. solanacearum* chromosome at a single *att*Tn*7* site (25-bp downstream of the *glmS* gene) and was confirmed by colony PCR.

### β-Galactosidase Assay

The gene expression level was evaluated by a *lacZYA* fusion-based β-galactosidase assay, and the assay (both *in vitro* and *in planta*) was performed as previously described (Zhang et al., 2013). Each assay was repeated for at least three independent experiments, and the averages with their respective standard deviation (SD) were determined. The statistical significance was assessed using the two-tailed Student’s *t*-test.

### qRT-PCR Analysis

Total RNA was isolated from the *hrp*-inducing medium cultured *R. solanacearum* cells using the TRIzol reagent according to the manufacturer’s instruction (Life, United States). After validating the RNA quality, cDNA was synthesized using the PrimeScript^TM^ RT Reagent Kit with gDNA Eraser (Perfect for Real Time, TAKARA, Japan). The One Step SYBR^®^ PrimeScript^TM^ PLUS RT-PCR Kit (TAKARA, Japan) was used for the qRT-PCR reactions with an Applied Biosystems 7500 Real-Time PCR System (Life, United States) to quantify the cDNA level. In this study, RipB, RipD, RipE, RipO, RipR, RipTAL, and RipW were selected for mRNA quantification by qRT-PCR and the *serC* gene was selected for reference (Monteiro et al., 2012). The primers used in this study were used as previously described (Wu et al., 2015). The experiments were repeated from RNA isolation for at least three independent replicates, and each treatment included four replications. The mean values were averaged with the standard deviation (*SD*), and statistical significance was determined using the two-tailed Student’s *t*-test.

### Virulence Assay and HR Test

The virulence assay and HR test were performed as described previously (Yao and Allen, 2007) with some modifications (Zhang et al., 2013). Wilt-susceptible tomato plants (*Solanum lycopersicum* cv. Moneymaker) and tobacco plants (*Nicotiana tabacum* CV. Bright Yellow) were used for the virulence test by soil soak (with bacteria at a final concentration of 10^7^ cfu g^-1^ of soil) and petiole inoculation (dropped 2 μl of bacteria at 10^7^ cfu ml^-1^ onto the fresh-cut surface of petioles). Each experiment included at least 10 plants per treatment, and each assay was repeated at least in triplicate. Mean values were manipulated with standard deviation (*SD*), and their statistical significance was determined using the two-tailed Student’s *t*-test. *N. tabacum* BY was used for the HR test with leaf infiltration, and the symptom development of HR was recorded periodically. Each experiment was repeated for at least three independent replicates, and a representative result was presented.

### Bacterial Growth *in Planta*

Bacterial growth *in planta* was performed as described previously (Zhang et al., 2013). Two to 3-week-old tomato plants were inoculated with bacteria using petiole inoculation, and the stem tissues (5 cm above the soil and 1 cm in length) were removed at 4 dpi (wilt symptoms reached to 1–2) and 7 dpi (wilt symptoms reached to 3–4). The cells were collected from the plant stem tissues and quantified by dilution plating. Each experiment was repeated for at least three independent replicates, and the mean values and *SD* values were determined. The statistical significance was assessed using the two-tailed Student’s *t*-test.

## Results

### NpdA Is Important for the *popA* Expression in *R. solanacearum in Vitro*

**FIGURE 1:**
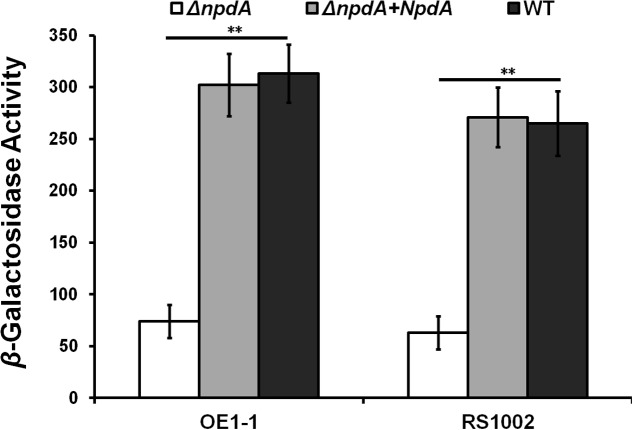
The *popA* expression in *Ralstonia solanacearum* strains. **Left**, OE1-1 derivatives; **right**, RS1002 derivatives. Black column, wild type; white column, the *npdA* deletion mutants; gray column, complementation with OE1-1 NpdA. Cells were grown in *hrp*-inducing medium to an OD_600_ of approximately 0.1 and subjected for β-galactosidase assay. The mean values of atleast 3 independent trials are presented in Miller units with SD (error bars). Significance level, ^∗∗^*P* < 0.01 (*t*-test).

The T3SS expression in OE1-1 was monitored with a *popA-lacZYA* fusion in our study. The *popA* expression in *npdA* transposon mutant was reduced to approximately 30% of that in the wild-type strain, suggesting that NpdA might be required for the T3SS expression in *R. solanacearum* OE1-1 (Zhang et al., 2013). To confirm this phenomenon, we created the *npdA* in-frame deletion mutant RK5628 (*popA-lacZYA*,Δ*npdA*) in OE1-1 strain and evaluated its *popA* expression level. First, no differences in the bacterial morphology and growth profiles were observed between the *npdA* mutants and wild-type strain in rich or *hrp*-inducing media (data not shown). Similar to that of the wild-type strain in rich medium, RK5628 did not express *popA*. However, in *hrp*-inducing medium, the *popA* expression in RK5628 was substantially reduced (an average of 74 vs. 313 Miller Units) (**Figure 1**). Additionally, the complemented *npdA* gene (integrated into the chromosome downstream of the *glmS* gene) could completely restore its *popA* expression to that of wild type in *hrp*-inducing medium (**Figure 1**), confirming that NpdA is an important factor for *popA* expression in *R. solanacearum* OE1-1. The strain RS1002 and GMI1000 exhibit distinctly different phenotype from OE1-1, and hence we generated the *npdA* deletion mutants from RS1002 and GMI1000. The *popA* expression was also substantially reduced in RS10013 (*npdA* deletion from RS1002) (**Figure 1**) and GF0021 (*npdA* deletion from GMI1000) (data not shown).

NpdA is highly conserved in *R. solanacearum* species, and through NCBI blast, the identities of NpdAs amino acids between 28 *R. solanacearum* strains are greater than 93%. For instance, there is only one residue different from the total 335 amino acids of NpdAs between OE1-1 and GMI1000. Furthermore, the *npdA* gene of OE1-1 could fully complement the *popA* expression in RS10013 to that of the wild-type strain (**Figure 1**), suggesting that the NpdA-dependent expression of *popA* is highly conserved in *R. solanacearum* species.

### NpdA Is Important for the Expression of Some T3Es in *R. solanacearum in Vitro*

**FIGURE 2:**
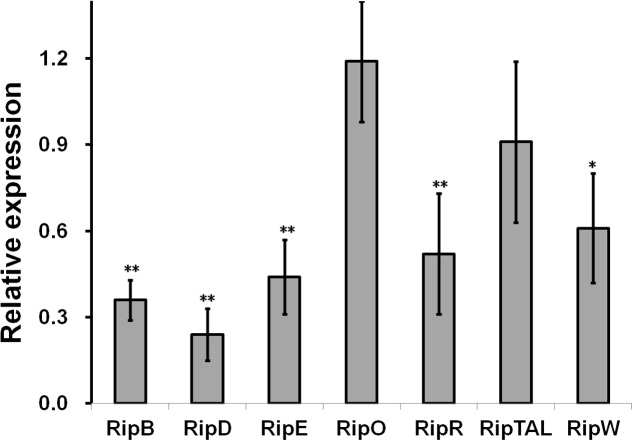
Relative expression of some T3Es genes in *R. solanacearum* OE1-1. Relative expression level of eight representative T3Es genes in *hrp*-inducing medium were determined by qRT-PCR and *serC* gene was used as reference gene. Normalized value was divided with that of wild type and relative values were presented. Each test was repeated in at least three independent trials and each trials included four replications. Mean values were averaged and presented with *SD* (error bars). Significance level, ^∗^*P* < 0.01, ^∗∗^*P* < 0.01 (*t*-test).

The *popA* gene exists as part of an operon with *popB* and *popC*, and belongs to T3Es. Since NpdA is important for the *popA* expression, we further investigated whether it was required for the expression of some other T3Es in OE1-1. In this study, RipB, RipD, RipE, RipO, RipR, RipTAL, and RipW were selected for mRNA quantification by qRT-PCR. Among these T3Es, the mRNA levels of RipB, RipD, RipE, and RipR in *hrp*-inducing medium were significantly reduced in RK5628 (*p* < 0.01), and the mRNA level of RipW was also distinctly reduced (**Figure 2**), (*p* < 0.05). However, the *npdA* deletion did not change the mRNA levels of RipO and RipTAL, suggesting that the NpdA is also important for the expression of a subset of T3Es in *R. solanacearum* OE1-1.

### NpdA Is Particularly Required for the Full Virulence of *R. solanacearum* OE1-1

**FIGURE 3:**
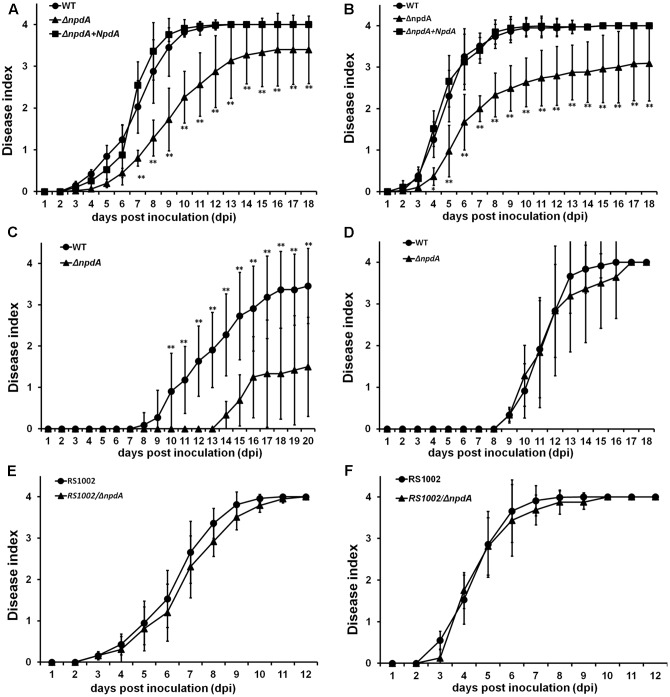
Pathogenicity test. *R. solanacearum* strains, **(A–D)** OE1-1 derivatives; **(E,F)** RS1002 derivatives. Inoculation methods: **(A,C,E)** soil soak inoculation, a bacterial suspension was poured into the soil of plants at a final concentration of 10^7^ cfu g^-1^ of soil; **(B,F)** petiole inoculation, about 3 μl of bacterial suspension at 10^8^ cfu ml^-1^ was dropped onto freshly cut surface of petioles; **(D)** Leaf infiltration in tobacco leaves, about 50 μl of bacterial suspension at 10^8^ cfu ml^-1^ was infiltrated into tobacco leaves with a blunt-end syringe. Plants, **(A,B,E,F)** tomato plants; **(C,D)** tobacco plants. Closed circle, wild type; closed triangle, *npdA* mutant; closed square, complementation with OE1-1 NpdA. Plants were inspected daily for wilt symptoms, and scored on a disease index scale from 0 to 4 (0, no wilting; 1, 1–25% wilting; 2, 26–50% wilting; 3, 51–75% wilting; and 4, 76–100% wilted or dead). Each assay was repeated in four independent trials (each trial contains at least 10 plants) and mean values were averaged and presented with *SD* (error bars). Significance level, ^∗^*P* < 0.01, ^∗∗^*P* < 0.01 (*t*-test).

The T3SS and some T3Es are important for the pathogenicity of *R. solanacearum* in host plants. Thus, we evaluated whether NpdA was required for the virulence of *R. solanacearum*. When tomato plants were challenged with soil-soak and petiole inoculation, RK5628 could wilt and kill most of the test plants, while approximately 25% of the test plants did not die even up to 18 dpi (days post inoculation). For example, 22 of the 96 total soil-soak inoculated tomato plants began to wilt at 8–10 dpi, but the wilt symptoms remained up to 18 dpi; then, the disease index of RK5628 was found to be approximately 3 at 12–18 dpi (**Figure 3A**). When tomato plants were challenged with petiole-inoculation, the virulence of RK5628 was also significantly reduced (**Figure 3B**). RK5628 exhibited significantly lower virulence than that of OE1-1 strain in tomato plants. With complementation, the *npdA* gene could completely restore its less virulence to that of wild type (**Figures 3A,B**), confirming that NpdA is important for the full virulence of *R. solanacearum* OE1-1 in tomato plants.

*Ralstonia solanacearum* OE1-1 is also virulent in tobacco plants. Thus, we evaluated whether NpdA was required for the virulence of OE1-1 in tobacco plants. When tobacco plants were challenged with soil-soak inoculation, significantly reduced virulence by RK5628 was also observed (**Figure 3C**). When the bacteria were directly inoculated into tobacco plants with leaf infiltration, no difference in virulence was observed between the RK5628 and OE1-1 (**Figures 3D**).

The RS1002 strain is virulent in tomato but elicits HR in tobacco leaves. We also tested the virulence of RS10013 (*npdA* deletion in RS1002 strain) in tomato plants using both the soil-soak and petiole inoculation methods, respectively. It was very intriguing to find that this *npdA* mutant exhibited almost identical virulence as that of the wild-type RS1002 strain (**Figures 3E,F**), suggesting that NpdA was not involved in virulence of RS1002 in host plants. The involvement of NpdA in virulence of *R. solanacearum* is specific to OE1-1 in host plants.

### NpdA Is Not Involved in the HR Elicitation of *R. solanacearum*

**FIGURE 4:**
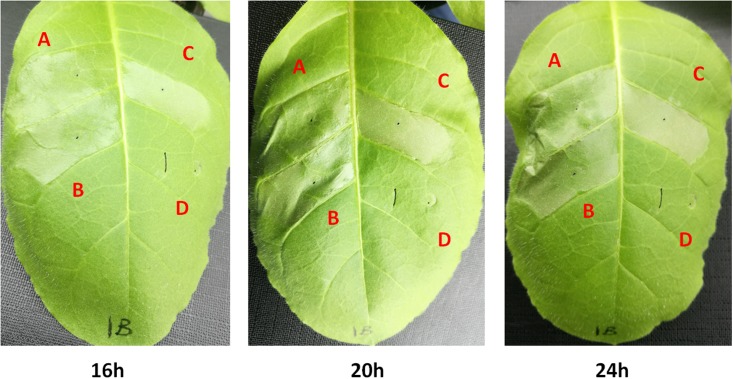
HR test. Approximate 50 μl of bacterial suspension at 10^8^ cfu ml^-1^ was infiltrated into the leaf mesophyll tissue with a blunt-end syringe. (A) RS1002; (B) *npdA* mutant (RK10013); (C) RK10015 (complementation with OE1-1 NpdA); (D) distilled water. Development of necrotic lesions was observed periodically and pictures were taken. Each experiment were repeated in triple and each treatment contains four plants. The results presented are from a representative experiment, and similar results were obtained in all other independent experiments.

Some T3Es are responsible for the HR elicitation in resistant plants. Thus, we evaluated whether NpdA is involved in the HR elicitation of RS1002 and GMI1000 in tobacco leaves. When infiltrated into tobacco leaves (*N. tabacum* BY), RS10013 (*npdA* deletion in RS1002 strain) caused apparent necrotic lesions at 16 hpi (hours post inoculation), and the area of necrotic lesions reached a maximum at 24 hpi (**Figure 4**). The *npdA* mutant in RS1002 caused almost the same development of necrotic lesions as RS1002 in tobacco leaves (**Figure 4**). Moreover, GF0021 (*npdA* deletion in GMI1000 strain) caused identical development of necrotic lesions as GMI1000 in tobacco leaves (data not shown), suggesting that NpdA was not involved in the HR elicitation of *R. solanacearum* strains in tobacco leaves.

### NpdA Is Not Required for the Growth of *R. solanacearum in Planta*

**FIGURE 5:**
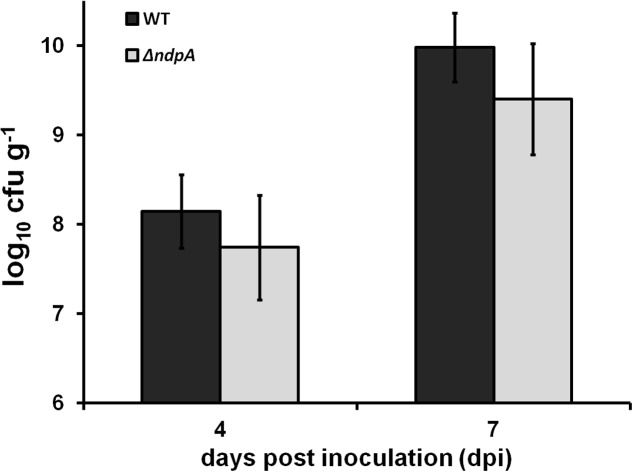
Bacterial growth test *in planta*. Tomato plants were inoculated with bacteria using petiole inoculation and stem species were removed at 4 and 7 dpi. Cells number was quantified by dilution plating. Black column, wild type (RK5050); gray column, *npdA* mutant (RK5628). Each experiment was repeated in triple and each treatment contains four plants. Mean values were averaged and presented with *SD* (error bars).

As a vascular pathogen, the growth capacity of *R. solanacearum* in host plants is essential for its virulence. Therefore, we evaluated whether NpdA was required for the growth of *R. solanacearum in planta*. We inoculated tomato plants with RK5628 using petiole inoculation and quantified the number of cells in stem tissues with dilution plating. RK5628 proliferated in the stem to approximately 10^8^ cfu g^-1^ at 4 dpi and approximately 10^10^ cfu g^-1^ at 7 dpi (**Figure 5**). RK5628 exhibited almost the same bacterial growth as that of OE1-1 in tomato stem, suggesting that NpdA was not required for the bacterial growth of *R. solanacearum in planta*.

### NpdA Is Important for the *popA* Expression *in Planta* But Only in OE1-1 Strain

**FIGURE 6:**
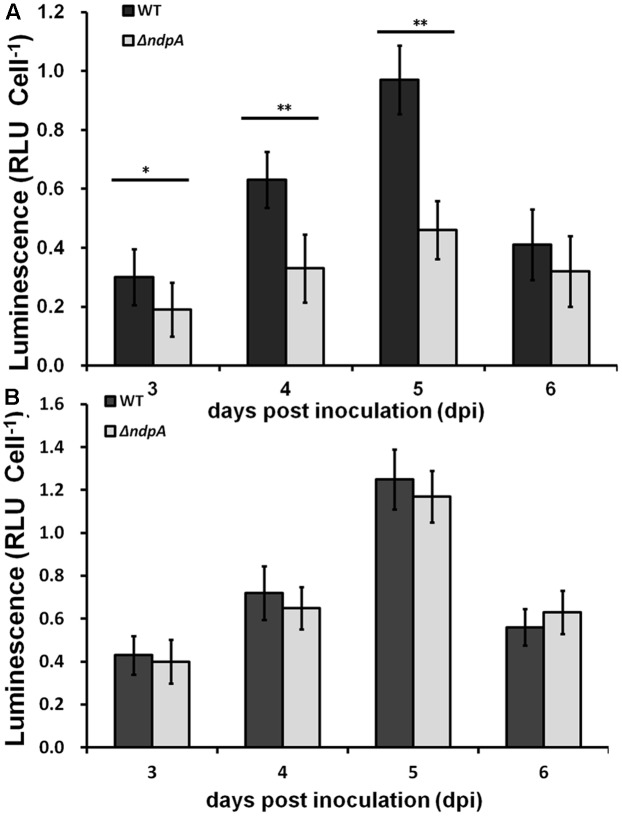
The *popA* expression *in planta*. **(A)** OE1-1 derivatives; **(B)** RS1002 derivatives. Black column, wild type strains; gray column, *npdA* mutants. Tomato plants were inoculated with bacteria using petiole inoculation and stem pecies were removed at 3–6 dpi for β-galactosidase assay *in planta* with Galacto-Light Plus kit and cells numbers were quantified by dilution plating. Each experiment was repeated at least in triple and each treatment contains four plants. Mean values were averaged and presented with *SD* (error bars). Significance level, ^∗^*P* < 0.01, ^∗∗^*P* < 0.01 (*t*-test).

The expression level of T3SS *in planta* could be induced to a much-higher level than that in the *hrp*-inducing medium. Thus, we further investigated whether NpdA was involved in the induction of T3SS expression in host plants. We challenged tomato plants with petiole-inoculation and collected the bacterial cells from the stems for the β-galactosidase assay *in planta* (normalized by cells number). The *popA* expression in the RK5050 (derivatives of OE1-1) increased at 3, 4, and 5 dpi and then decreased quickly to a much lower level at 6 dpi (Zhang et al., 2013). We detected their expression daily from 3 dpi (the wilt symptom appeared) to 6 dpi (wilt symptom increased to 3–4). Similar to that of wild type, the *popA* expression of RK5628 (derivatives of OE1-1) in tomato stems increased from 3 dpi, reached a maximum at 5 dpi, and then quickly decreased at 6 dpi (**Figure 6A**). It was distinctly less than that of the wild-type strain in tomato stems at 3 dpi (*p* < 0.05) and significantly less than that of the wild-type strain at 4–5 dpi (*p* < 0.01), suggesting that NpdA is also important for the *popA* expression of *R. solanacearum* OE1-1 in tomato plants. Although RK10013 (derivatives of RS1002) exhibited identical virulence to wild type RS1002, we also evaluated the *popA* expression in RK10013 in tomato stems. It was intriguing that RK10013 exhibited almost identical *popA* expression in tomato stems to that of wild type RS1002 (**Figure 6B**), indicating that the NpdA was not required for the *popA* expression of *R. solanacearum* RS1002 in tomato stems. All these suggested that the NpdA-dependent *popA* expression *in planta* was specific to OE1-1 strain.

### NpdA Affected the T3SS Expression Through HrpB But Was Independent of Other Regulators

**FIGURE 7:**
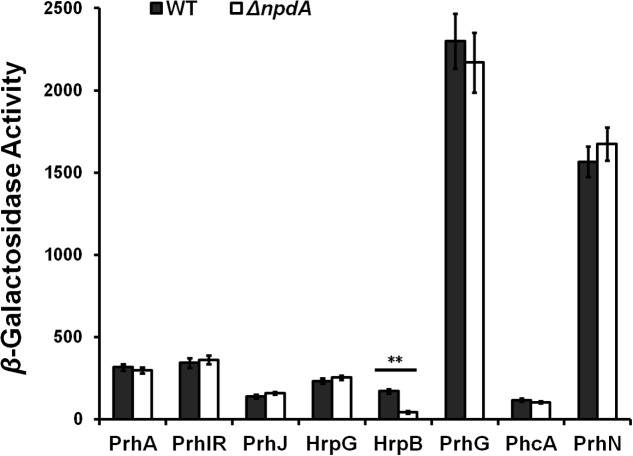
Expression of some known regulators in *R. solanacearum.* Black column, wild type (RK5050); gray column, *npdA* mutant (RK5628). Cells were grown in *hrp*-inducing medium to an OD_600_ of approximately 0.1 and subjected for β-galactosidase assay *in vitro*. Mean values were averaged and presented with *SD* (error bars). Significance level, ^∗∗^*P* < 0.01 (*t*-test).

*Ralstonia solanacearum* uses HrpB to directly control the expression of the entire T3SS and T3Es, and the *hrpB* expression is regulated by a complex regulation network. We therefore investigated whether NpdA affected the T3SS expression through some known pathway. We generated some *npdA* deletion mutants from OE1-1 derivatives (**Table 1**) and evaluated its effect on the expression of these known regulators, including the *prhA*, *prhIR*, *prhJ*, *hrpG*, *prhG*, *hrpB*, *phcA*, and *prhN* genes. The deletion of *npdA* substantially reduced the *hrpB* expression in *hrp*-inducing medium (43 vs. 175 miller units), while it did not change the expression of other regulators, either in *hrp*-inducing medium (**Figure 7**) or rich medium (data not shown), suggesting that NpdA affected the T3SS expression through HrpB and its influence was independent of these known regulators.

## Discussion

In the present study, we demonstrated that *R. solanacearum* NpdA was involved in the infection process of OE1-1 in host plants. NPDs exist extensively in many microorganisms and plants. In plants, NPDs are reported to be defense or stress-related enzymes. For example, *npdA* is significantly upregulated in hypocotyl tissues once the cotton seedling is infected with *Fusarium oxysporum* f. sp. *vasinfectum* (Dowd et al., 2004). NPDs are a type of oxidoreductases, which catalyze 2-nitropropane into nitrite and acetone, and participate in nitrogen metabolism (Kido et al., 1976; Francis et al., 2005). Moreover, they exhibit potential abilities in detoxification and bioremediation since they can also degrade nitroalkanes and some nitro-aromatics, which are usually toxic to microorganisms or harmful to the environment, into nitrate and nitrite (Nishino et al., 2010). Plants produce many organic compounds, and hence, their interacting bacteria should have evolved traits to degrade these metabolites. Comparative genome analysis revealed that a plant-associated bacterial endophyte *Burkholderia phytofirmans* PsJN evolved a high number of ring cleavage enzymes, including the 2-nitropropane dioxygenase, to degrade plant-produced organic compounds (Mitter et al., 2013). The genome analysis also reveales that SAR86, an abundant but uncultivated marine bacterial lineage, harbors 7 nitropropane dioxygenases for antibiotic resistance (Dupont et al., 2012). However, up to date, there are barely reports that show that NPD is related to the pathogenicity of some pathogens.

T3SS plays an essential role in *R. solanacearum* virulence, and in this study, NpdA was demonstrated to be important for T3SS expression in *R. solanacearum*. NpdA is highly conserved in *Ralstonia* species and other bacteria, and NpdA of OE1-1 could indeed act on the *popA* expression in RS1002 strain and could completely substitute that of RS1002 in *hrp*-inducing medium. The NpdA-dependent expression of T3SS expression in *hrp*-inducing medium is universal in *R. solanacearum* species. *R. solanacearum* generally invades host plants from the root, multiplies rapidly in the root cortex, and then finally enters into xylem vessels (Roberts et al., 1988; Vasse et al., 1995). However, the deletion of *npdA* in RS1002 strain did not change its virulence in tomato plants, suggesting that the NpdA was specifically required for the virulence of *R. solanacearum* OE1-1 in host tomato plants.

As a vascular plant pathogen, the extensive proliferation in the host xylem and abundant production of extracellular polysaccharides (EPS) are also regarded as the main factors in the virulence of *R. solanacearum* (Denny, 1995). The *npdA* mutants exhibited almost the same bacterial morphology and growth profiles as OE1-1 strain in the media and tomato stems, suggesting that the growth defects in host plants were not clues of reduced virulence caused by *npdA* deletion in OE1-1. The deletion of *npdA* substantially reduced the T3SS expression of *R. solanacearum* OE1-1, both *in vitro* and *in planta*. Moreover, the *npdA* deletion also substantially reduced the expression of a subset of T3Es of OE1-1 that plays an important role in the interaction between *R. solanacearum* and host cells, indicating that the involvement of NpdA in virulence of OE1-1 should be due to its impaired expression of T3SS and T3Es.

When the tobacco plants were challenged, the *npdA* mutant of OE1-1 exhibited significantly reduced virulence than that of the wild-type strain but only when the tobacco plants were inoculated with soil soak inoculation, which mimics the natural invasion of *R. solanacearum* through the roots (Valls et al., 2006). When the tobacco plants were inoculated by leaf infiltration, in which the bacteria directly invaded the host plants, the *npdA* mutants of OE1-1 exhibited identical virulence as that of the wild-type strain, indicating that NpdA might function mainly at the early stage of infection process of OE1-1 in tobacco plants. It was consistent with previous reports that some regulators might play different roles at different infection stages, e. g., the PrhG regulator might play an important role in the late stage of infection in the tomato stem (Zhang et al., 2013). Although expression of the T3SS in *npdA* mutant of RS1002 was also significantly reduced in *hrp*-inducing medium, the *npdA* deletion did not affect the T3SS expression of RS1002 in tomato stems, suggesting the NpdA was not required for the T3SS expression of RS1002 *in planta*. This could explain why the *npdA* deletion did not affect the virulence of RS1002 in tomato plants with both soil-soak and petiole inoculation, and why the *npdA* deletion did not delay the HR elicitation of RS1002 in tobacco leaves. The NpdA should function in virulence differently among *R. solanacearum* strains, and this was consistent with the characteristics of the complex *R. solanacearum* species.

The expression of T3SS and T3Es are directly controlled by the master regulator HrpB (Arlat et al., 1992; Genin et al., 1992; Vasse et al., 2000; Mukaihara et al., 2004, 2010), and *hrpB* expression is positively regulated by HrpG and PrhG in a parallel way (Plener et al., 2010; Zhang et al., 2013). The activation of *hrpB* expression needs host plant signals or some mimic signals, and these signals are integrated with HrpG and PrhG, which respond to these signals by their phosphorylation (Yoshimochi et al., 2009b). The *npdA* deletion significantly reduced the *hrpB* expression, which was consistent with the fact that HrpB directly controls the expression of the T3SS and T3Es. However, deletion of *npdA* did not change the expression of *hrpG* and *prhG*, and NpdA was independent of the expression of some other regulators. NpdA should affect the expression of the T3SS through HrpB in some novel pathway.

Taken together, the NpdA was demonstrated to be important for the T3SS expression in *R. solanacearum* species, but it was specificantly important for the the T3SS expression *in planta* and full virulence of OE1-1 in host plants. NpdA affected the T3SS expression mediated with HrpB but through some novel pathway. This is the first study that indicates NPDs could be involved in the pathogenicity of pathogens. This finding will help us further investigate the complex regulation of the pathogenesis of *R. solanacearum* and provide new insights into the functions of NPDs.

## Author Contributions

YZ and KO conceived and designed the experiments. WZ, JL, YT, and YC performed the experiments. XS, KO, and YZ analyzed and discussed the results. YZ wrote and revised the paper.

## Conflict of Interest Statement

The authors declare that the research was conducted in the absence of any commercial or financial relationships that could be construed as a potential conflict of interest.
